# The electro-optic mechanism and infrared switching dynamic of the hybrid multilayer VO_2_/Al:ZnO heterojunctions

**DOI:** 10.1038/s41598-017-04660-2

**Published:** 2017-06-30

**Authors:** Peng Zhang, Wu Zhang, Junyong Wang, Kai Jiang, Jinzhong Zhang, Wenwu Li, Jiada Wu, Zhigao Hu, Junhao Chu

**Affiliations:** 1 Key Laboratory of Polar Materials and Devices (MOE) and Technical Center for Multifunctional Magneto-Optical Spectroscopy (Shanghai), Department of Electronic Engineering, East China Normal University, Shanghai, 200241 China; 2 Department of Optical Science and Engineering, Fudan University, Shanghai, 200433 China

## Abstract

Active and widely controllable phase transition optical materials have got rapid applications in energy-efficient electronic devices, field of meta-devices and so on. Here, we report the optical properties of the vanadium dioxide (VO_2_)/aluminum-doped zinc oxide (Al:ZnO) hybrid *n-n* type heterojunctions and the corresponding electro-optic performances of the devices. Various structures are fabricated to compare the discrepancy of the optical and electrical characteristics. It was found that the reflectance spectra presents the wheel phenomenon rather than increases monotonically with temperature at near-infrared region range. The strong interference effects was found in the hybrid multilayer heterojunction. In addition, the phase transition temperature decreases with increasing the number of the Al:ZnO layer, which can be ascribed to the electron injection to the VO_2_ film from the Al:ZnO interface. Affected by the double layer Al:ZnO, the abnormal Raman vibration mode was presented in the insulator region. By adding the external voltage on the Al_2_O_3_/Al:ZnO/VO_2_/Al:ZnO, Al_2_O_3_/Al:ZnO/VO_2_ and Al_2_O_3_/VO_2_/Al:ZnO thin-film devices, the infrared optical spectra of the devices can be real-time manipulated by an external voltage. The main effect of joule heating and assistant effect of electric field are illustrated in this work. It is believed that the results will add a more thorough understanding in the application of the VO_2_/transparent conductive film device.

## Introduction

Narrow band gap vanadium dioxide (VO_2_) has attracted intensely studied in recent years because of the unique properties of metal-insulation transition (MIT) at a temperature of about 68 °C^1, 2^. The optical properties of the VO_2_ film change abruptly at the infrared range and the resistivity decreases more than four orders with the occurrence of the MIT. The lattice in the metallic phase has the rutile structure (*P*4_2_/*mn*), with the vanadium ions arranged in periodic chains parallel to the *c*-axis. In the insulating phase, the dimerization and the off-axis zigzag displacement of V-V pairs can result in a monoclinic structure (*P*2_1_/*c*)^3, 4^. The MIT can be regulated by multiple ways, such as mechanical strain^5–8^, photocarrier or chemical doping^9–12^, stoichimetric ratio of V/O^13, 14^, and electric field^15–17^. These prominent optical and electrical features have sparked numerous potential applications in efficient smart windows^18, 19^, optoelectronic switch^20, 21^ and field-effect transistors^22, 23^. However, due to the high phase transition temperature (*T*_MIT_), the practical devices have not been developed widely. Therefore, more comprehensive understanding of VO_2_ properties should be added in the tuning of the MIT process for more reliable applications.

As the heterostructure related to VO_2_ film was often applied to construct the functional devices and regulate the optical and electrical properties, much attention has been pay for to it. It was found that the localized surface plasmon resonances and optical memory effect can be modulated by the hybrid nanoscale devices of Au nanostructures-VO_2_ film^24^. The hybrid Au-VO_2_-SiO_2_-Si heterojunction was used to monitor the electrical switching in VO_2_ by Markov *et al*.^25^. Kim *et al*. utilized the hybridized large-area graphene-supported VO_2_ structure to manipulate the switching behavior and *T*_MIT_^26^. Few-layer graphene is recognized as a transparent conductive film (TCF), the optical response of VO_2_ can be enhanced and the *T*_MIT_ can be reduced when VO_2_ film grown on it^27, 28^. Gao *et al*. utilized the Al:ZnO as the capping layer and FTO as buffer layer to study the optical mechanism of the VO_2_/Al:ZnO and VO_2_/FTO structures^29, 30^. The dynamic infrared modulation of the VO_2_/ITO device was reported by Li *et al*.^31^. This indicates that the transition behavior can be tuned by varying the transparent conductive buffer layer, such as graphene, Al:ZnO, FTO and ITO. Though few-layer graphene is a prominent flexible substrate, the transfer method is circuitous. In addition, the conductivity and transmittance of FTO is inferior than those of ITO. It can be seen that the ITO film was often used to drive the phase transition of VO_2_ by joule heating in the system of VO_2_/ITO device. However, the properties of ITO film will be degenerated in the case of high annealing temperature. On the contrary, Al:ZnO, a promising transparent conductive film, the electrical and optical properties of which can be comparable to those of the ITO film. The transmittance, tuned by voltage in VO_2_/Al:ZnO system has not been reported previously. Therefore, it is necessary to investigate the fundamental optical and electrical properties of the hybrid multilayer structures systematically. Also, more complicated device based on the hybrid multilayer heterojunctions is needed to provide deeper understanding on the application of the VO_2_/TCF device.

In this article, we report a successful preparation of VO_2_/Al:ZnO hybrid multilayer heterostructure by pulsed laser deposition (PLD). Various structures are constructed to compare the difference of the optical and electrical properties. We discuss various optical responses of the hybrid material as a function of the number of Al:ZnO layers and temperature. It is found that the reflectance spectra at infrared region present the wheel phenomenon with temperature due to the surface interference. The Fabry-Perot-type effect, which takes constructive and destructive interference of the optical thin films is shown at near-infrared range. In addition, the abnormal Raman vibration mode is found in the double layer Al:ZnO structure at the insulator region, which is different from the usual Raman mode in pure VO_2_ film. Different from the previous device, the ladder-like electrode was adopted to verify the main effect of the joule heating and assistance effect of electric field in present study. The electro-optic performance of each device was shown and compared by adding the external voltage.

## Results and Discussion

### Microstructure analysis

**Figure 1:**
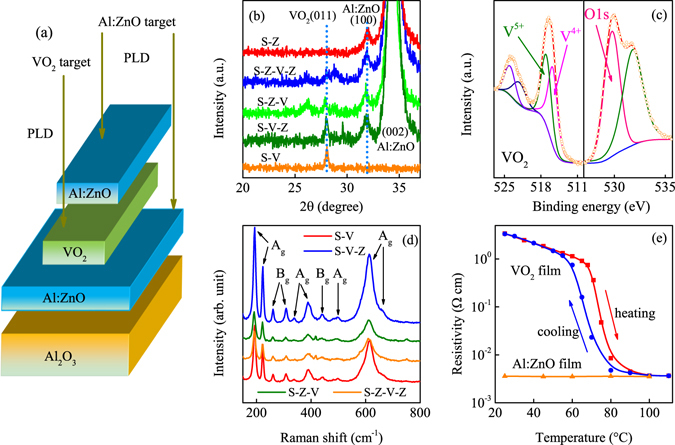
(**a**) The detailed structure of the hybrid S-Z-V-Z heterojunction. (**b**,**c**) The XRD curves of the five structures and the XPS spectra of pure VO_2_ film, respectively. (**d**) The Raman spectra of the hybrid structures (**e**) The resistivity of the VO_2_ and Al:ZnO film with temperature, respectively.

Figure 1(a) shows the hybrid multilayer sandwich structure. The Al_2_O_3_ substrate, Al:ZnO and VO_2_ were abbreviated by S, Z and V to make the written more concisely. Then, the expression of the structure in Fig. 1(a) can be simplified by S-Z-V-Z. The other structure was Al_2_O_3_/VO_2_ (S-V), Al_2_O_3_/Al:ZnO (S-Z), Al_2_O_3_/Al:ZnO/VO_2_ (S-Z-V) and Al_2_O_3_/VO_2_/Al:ZnO (S-V-Z), respectively. (Supplementary Information S1) It should be emphasized that the growth times of the VO_2_ and Al:ZnO film are 30 min and 60 min for all the five structures, respectively. The thickness of the Al:ZnO should be nearly the same for the structures, as well as the VO_2_ film. Therefore, the thickness variation of the VO_2_ and Al:ZnO film is subtle for all five structures. The thicknesses of the VO_2_ and Al:ZnO film are about 50 nm and 140 nm for the five structures, respectively. The section images can be seen in Supplementary Information S2. The XRD patterns of VO_2_/Al:ZnO systems are shown in Fig. 1(b). It can be seen that the peaks located at 28.06°, 28.01°, 27.93° and 28.01° are assigned to the S-V, S-V-Z, S-Z-V and S-Z-V-Z, respectively. If the *T*_MIT_ of VO_2_ is modulated by the strain between the interface of VO_2_ and Al:ZnO, the peak value will increase or decrease monotonously with the strain^6, 32, 33^. It can be seen that the *T*_MIT_ is 70 °C, 64.1 °C, 63.8 °C and 61 °C for S-V, S-V-Z, S-Z-V and S-Z-V-Z, respectively. However, the peak value of the VO_2_ film is nearly the same and not increase or decrease monotonously, which indicates that the strain state four structures are similar and the strain is relaxed fully. Therefore, it can be concluded that the effect of strain is subtle and can be ignored. Note that the XRD peaks of VO_2_ are relative weak, which can be attributed to the film thickness or the effect of the double layer Al:ZnO. The 2θ peak at about 31.93° and 34.41° can be assigned to Al:ZnO (100) and Al:ZnO (002) diffractions, respectively. Figure 1(c) shows the V_2*p*_ and O_1*s*_ photoelectron spectra of VO_2_ film deposited on Al_2_O_3_ substrate. The chemical element of C1s with binding energy of 285 eV was used to calibrate the spectra. The peaks at 515.8 eV and 517.1 eV are assigned to V 2*p*_3/2_, which represents V^4+^ and V^5+^, respectively^18, 34^. The peaks at 522.3 and 523.9 eV are attributed to V 2 *p*_1/2_ core level. The main peak and additional shoulder peak located at about 529.9 eV and 531.8 eV can be assigned to O 1 *s* and the surface adsorption oxygen, respectively^35^. The XPS spectra of Al:ZnO film and the detailed analysis are shown in Supplementary Information S3. The Raman spectra of the hybrid structures were presented in Fig. 1(d). The M _1_ phase shows the scattering peak at 193 (A_*g*_), 221 (A_*g*_), 260 (B_*g*_), 310 (B_*g*_), 339 (A_*g*_), 392 (A_*g*_), 442 (B_*g*_), 496 (A_*g*_), 614 (A_*g*_) and 665 cm^−1^ (A_*g*_), consistenting with previous reports^36, 37^. Figure 1(e) shows the temperature dependent resistivity curves of VO_2_ and Al:ZnO films. The resistivity drop for the VO_2_ film is about three orders while the resistivity of the Al:ZnO film is independent with temperature. The resistivity of the Al:ZnO film is close to 10^−3^ Ω cm, indicating that the Al doping improves the conductivity of the ZnO film.

### NIR-UV transmittance

**Figure 2:**
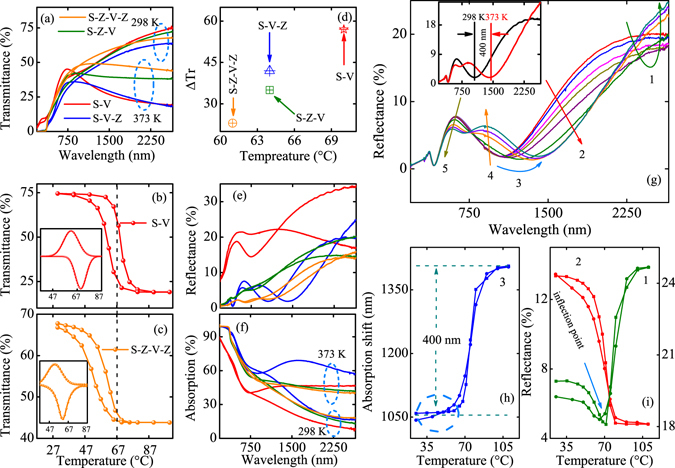
(**a**–**c**) The low and high temperature transmittance, hysteresis loops and differential curves for the structures. (**d**) The relationship of the *T*_MIT_ and ΔTr. (**e**,**f**) The low and high temperature reflectance and absorption. (**g**) The temperature dependent reflectance for the S-V-Z. The inset is the red-shift of the wave. (**h**,**i**) The hysteresis loop of the region marked by 1–3 in figure (**g**).

Figure 2(a) shows the low temperature and high temperature transmittance of the four hybrid multilayer heterojunctions. The detailed temperature dependent transmittance can be seen in Supplementary Information S4. The infrared transmittance changes at *λ* = 2650 nm are 56.4%, 45.6%, 34.3% and 23.9% for S-V, S-V-Z, S-Z-V and S-Z-V-Z, respectively. The parameters of the optical properties were shown in Table 1. The ΔTr, ΔR and ΔA represent the variation of the transmittance, reflectance and absorption with the temperature at 2650 nm, respectively. The phase transition temperature and full width at half maximum of differential was denoted by T_*c*_ and F, respectively. The discrepancy of the T_*c*_ between the heating and cooling process was defined by Δ*T*_*c*_. It can be seen that the change of transmittance is reduced due to the effect of Al:ZnO film. It was found that the variation of the transmittance is distinct from the effect of the buffer Al:ZnO layer and capping Al:ZnO layer. The effect of the capping Al:ZnO layer to optical properties is much weaker than that for the buffer layer. We believe that the discrepancy is closely related to the interface contact situation of the VO_2_ and Al:ZnO film. We found that the totally effect of the single buffer layer or the capping layer is equal to the influence of the double Al:ZnO layer. Comparing to the ΔTr of the S-V, the ΔTr is decreased by 10.8 and 22.1 for the S-V-Z and S-Z-V, respectively. The sum of the decreased ΔTr is 32.9, which is identical to the decreased ΔTr (32.5) of the S-Z-V-Z. The results demonstrate the interface contact condition of the two film resulted in the transmittance discrepancy indeed. Figure 2(b) and (c) show the hysteresis loops of transmittance at 2650 nm and the related differential curves. From the differential curves, the *T*_MIT_ is 70 °C and 61 °C for S-V and S-Z-V-Z, respectively. The detailed comparison of the hysteresis loops and differential curves of the four structures were shown in Supplementary Information S5. For the structure of S-V-Z, S-Z-V, the *T*_MIT_ was reduced by 4 °C compared to bulk VO_2_ film. We found that the *T*_MIT_ was decreased by 7 °C for the S-Z-V-Z, nearly two times than that for the single layer Al:ZnO film. Figure 2(d) presents the relationship between the *T*_MIT_ and ΔTr. It can be seen that the decreased *T*_MIT_ will sacrifice the variation of the transmittance. The phenomenon is similar to the metal ion doping^38–40^. The internal action mechanism will be discussed below.

**Table 1:** The parameters of the optical properties of the hybrid heterojunctions extracted from the related differential curves fitted by Gaussian function.

Samp.	T_*c*_ (°C)	F (°C)	Δ*T*_*c*_ (°C)	ΔTr	ΔR	ΔA
S-V	70.3	8.8	8.4	56.4	17.5	38.9
S-V-Z	64.1	14.2	9.2	45.6	5.5	40.6
S-Z-V	63.8	17.2	4.1	34.3	5.1	28.8
S-Z-V-Z	61.0	12.4	8.4	23.9	1.8	22.2

### Reflectance and absorption

For further observing the spectra discrepancy, the low and high temperature reflectance spectra were measured and shown in Fig. 2(e). The reflectance variation of the S-V-Z was shown in the inset of Fig. 2(g). It is worthy noting that the variation of the reflectance is less than 6% for the S-V-Z, S-Z-V and S-Z-V-Z. However, the change of the reflectance is more than 17% for the S-V. Then, the absorption spectra were obtained according to the relationship of T + R + A = 1. It is found that the variation of the absorption is dramatic than that for the reflectance. The variations of the absorption are about 39%, 41%, 29% and 22% for the S-V, S-V-Z, S-Z-V and S-Z-V-Z, respectively. From the change of the absorption, we can see that the maximum absorptivity occurs in the structure of S-V-Z, which indicates that the effect of the buffer layer and the capping layer to optical properties of VO_2_ film has a large discrepancy. It can be seen that the variation trend of the reflectance spectra of S-V and S-Z-V is similar. However, it is surprised that the change of the reflectance is totally different from the other two structures, especially for the S-V-Z, which was shown in Fig. 2(g). The spectra were divided by five parts and marked by 1–5. First, it shows that the reflectance at part 1 presents the wheel phenomenon, which decreases and then increases. The hysteresis loops of part 1 and 2 at 2650 nm and 1770 nm were displayed in Fig. 2(i). For the hysteresis loop of 1, there is an inflection point at near 70 °C, before and after which, there are two transition process. Coincidentally, it was found that the transition process of 2 almost complete when the inflection point occurred. Further, there is a redshift of the valley from 1039 nm to 1404 nm with temperature and the redshift process was shown in Fig. 2(h). The phenomenon is similar to the Fabry-Perot-type effect, which was reported by Lei *et al*.^24^. It is worthy noting that the Fabry-Perot-type effect also occurred at about 70 °C, consistent with that for the inflection point. According to the phenomena, it is believed that strong interference and the dielectric function change of VO_2_ film promoted the redshift of the valley and the reflectance wheel.

**Figure 3:**
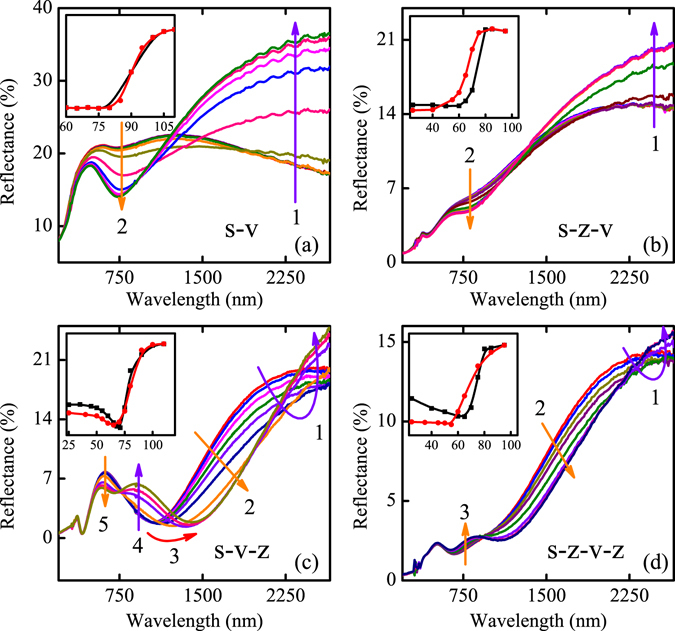
(**a**–**d**) The temperature dependent reflectance spectra for the four structures. The inset shows the variation of the reflectance with temperature at 2650 nm.

In order to rule out the effect of the thermal expansion between the interface of the two films, the detailed temperature dependent reflectance are shown in Fig. 3. From the curves in (a) and (b), the reflectance increases monotonously with temperature at infrared range and the redshift of the valley does not appear in the S-V and S-Z-V. The hysteresis loops of the reflectance at 2650 nm were shown in the inset of Fig. 3(a) and (b), which present the traditional hysteresis behavior. The result indicates that the contribution of the thermal expansion to the wheel of the reflectance can be ignored. It can be seen that the change of the reflectance is the part of infrared and visible region for (a) and (b), where the 1 and 2 were labeled. However, the variation of the reflectance mainly reflected by the region of 1–5 and 1–3 in Fig. 3(c) and (d), respectively. The changes of the reflectance at 2650 nm were presented in the inset of (c) and (d). It was shown that the two transition processes appeared due to the existence of capping Al:ZnO layer. It can be concluded that the effect of the buffer and capping layer of Al:ZnO to the VO_2_ film is different. The buffer layer of Al:ZnO acts as an ordinary substrate while the capping layer can be regarded as an optical coatings of transparent highly absorptive dielectric films.

The buffer layer has little contribution to the light interference whereas the capping layer constructed the light interference in the hybrid structure. The divergent change of the reflectance indicates that the Fabry-Perot type cavities established by buffer and capping layer are asymmetry. It has been reported that the absorption of the Fabry-Perot cavities constructed by Au/Ge shifts from about 520 nm to 820 nm^41^. In the present study, the absorption shifts from 1039 nm to 1404 nm, indicating the strong modulation of the near-infrared spectra. From Fig. 3(d), it was found that the redshift of the valley is unconspicuous for the structure with double layer Al:ZnO film, indicating the destructive interference effects of the buffer layer. It was believed that the localized surface-plasmon resonance and the controllable optical phase can be attributed to the complex refractive indices change of the films^24, 41, 42^. The variation of the optical spectra of Al:ZnO film is subtle and can be ignored. (Supplementary Information S6) Therefore, the complex refractive indices change of the VO_2_ film plays a main role in the redshift of the absorption.

**Figure 4:**
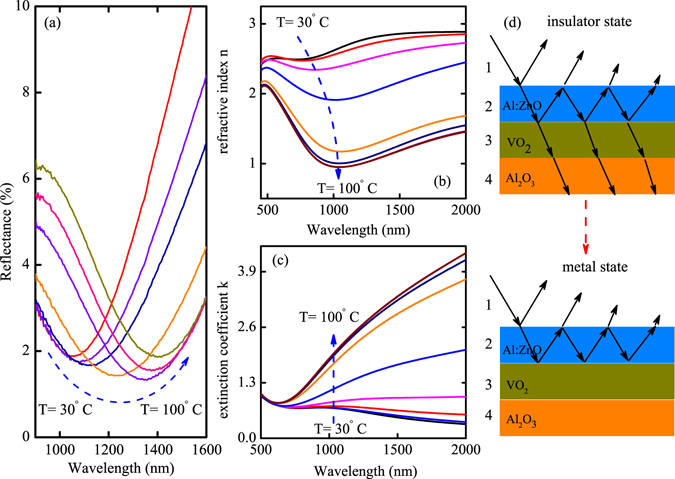
(**a**) The detailed red-shift of the absorption valley at the wavelength range of interest. (**b**,**c**) The temperature dependent of refractive index and extinction coefficient for the bare VO_2_ film, respectively. (**d**) The schematic of the Fabry-Perot type cavities comprising the insulator and metal state of VO_2_.

Figure 4(a) shows the temperature dependent wave shift. The valley red-shift from the wavelength at 1039 nm to the wavelength at 1404 nm, the discrepancy reaches 400 nm. It has been reported that the extinction peak blue shifts from 950 nm to 830 nm due to the localized surface-plasmon resonance effect^24^. In the present structure, one can see that the modulation of the valley is substantial due to the formation of the Fabry-Perot type cavities. The red-shift of the wave can be ascribed to the change of dielectric function, which are shown in Fig. 4(b) and (c). The dielectric function was extracted by fitting transmittance through Drude-Lorentz (DL) oscillator dispersion relation. It shows that the refractive index of VO_2_ gradual decreases and the valley red-shifts with temperature. Accordingly, the extinction coefficient increases with temperature in the near-infrared region. The change of the dielectric function accounts for the observed red-shift in the S-V-Z heterojunction. Different from the blue-shift phenomenon in Au hybrid structure^24, 41^, the transparent conducting oxide layer makes the absorption peak red-shift. Figure 4(d) shows the schematic of incident light from the structure comprising insulator state and metal state of VO_2_. One can see that the most of the light pass through the mediums when VO_2_ is in insulator state while the Fabry-Perot cavities are formed due to the VO_2_ film turns into the metal state.

### Temperature-dependent Raman spectra

**Figure 5:**
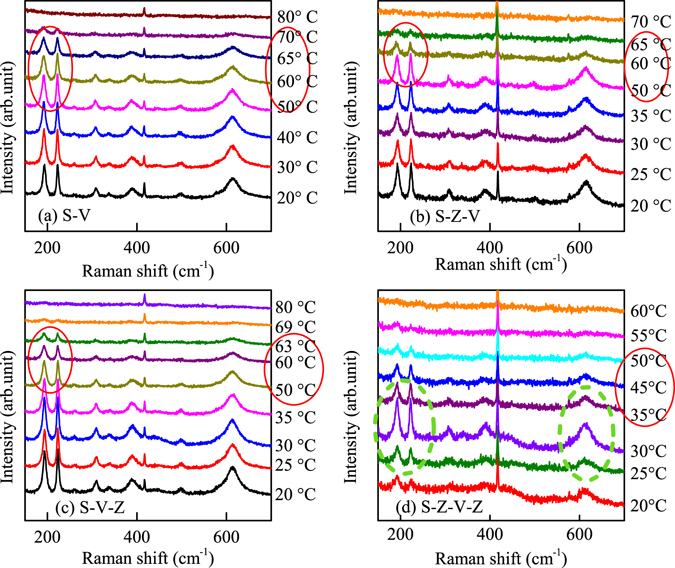
(**a**–**d**) The temperature-dependent Raman spectra for the four structures from 20 to 90 °C. The red solid and green dashed circles was used to display the transition process and the abnormal variation of Raman intensity.

**Figure 6:**
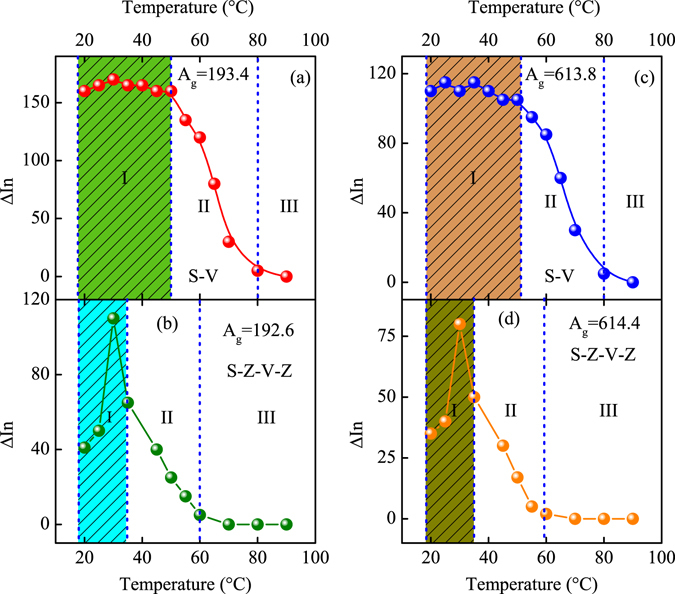
(**a**–**d**) The temperature-dependent relative Raman intensity for S-V and S-Z-V-Z, respectively. The Raman intensity of S-V follows the traditional behavior while S-Z-V-Z presents the abnormal variation.

To observe the variation of the Raman spectra for the structures, the temperature-dependent Raman spectra were shown in Fig. 5(a–d). The location of the Raman peak was marked above in Fig. 1(d), where the phonon modes can be uniquely assigned. The distinct peaks located at about 193, 221 and 614 cm^−1^ demonstrate that the VO_2_ films are of M _1_ phase at room temperature. From the Raman spectra, it can be seen that the phase transition temperatures are about 67 °C, 60 °C, 60 °C and 50 °C for S-V, S-Z-V, S-V-Z and S-Z-V-Z, respectively. The solid red circle was marked in the pictures to sign the phase transition process. The temperatures of phonon soften totally are about 80 °C, 70 °C, 69 °C and 60 °C for the four structures, respectively. The phase transition temperature of Raman is lower than that for the transmittance, especially for the S-Z-V-Z structure. This indicates that the phonon soften energy is reduced greatly due to the effect of the Al:ZnO film. Noted that Raman spectra presents the common transition behavior for the S-V, S-Z-V and S-V-Z structures. However, there is a distinct abnormal variation with temperature for the S-Z-V-Z Raman spectra, which is labeled by dashed green circle in Fig. 5(d). In order to rule out the coincidence factor, the Raman experiment was repeated several times. Whereas, the results are nearly the same, which indicates that the abnormal phenomenon exists indeed in the double layer Al:ZnO hybrid structure. To compare the discrepancy of the phonon mode, the relationship of the Raman relative intensity (ΔIn) with temperature was presented in Fig. 6(a–d) for S-V and S-Z-V-Z.

From Fig. 6(a), it is found that the A_*g*_ mode at 193.4 cm^−1^ presents the tradition hysteresis behavior for the pure VO_2_ film. The hysteresis loop was divided by three parts, which were marked by I, II and III. Unambiguously, the three parts present the insulator state, intermediate state and metal state, respectively. The change of ΔIn is within 10, which illustrates that the Raman activity remains unchanged in the insulator state. However, it is strange that the ΔIn increases greatly and then decreases with temperature at the insulator region I in Fig. 6(b). The change of the ΔIn for A_*g*_ mode at 192.6 cm^−1^ is about 70. To be the best of our knowledge, the abnormal phenomenon is rarely reported by previous literatures. It can be seen that the ΔIn is higher than that for the original state before 35 °C. Therefore, we defined the part before 35 °C as insulator region. To research the behavior of other phonon mode, the hysteresis loop of A_*g*_ mode at about 613.8 and 614.4 cm^−1^ was presented in Fig. 6(c) and (d), respectively. It can be seen that the behavior of A_*g*_ mode at 613.8 and 614.4 cm^−1^ in Fig. 6(c) and (d) is similar to that in Fig. 6(a) and (b). It is found that the ΔIn is higher than 100 for A_*g*_ mode at about 193 and 222 cm^−1^ at insulator state for S-V, S-Z-V and S-V-Z. However, the ΔIn is only 40 at 20 °C in Fig. 6(b). It seems that the Raman intensity was suppressed by the double layer Al:ZnO film. The phenomenon was puzzling so that the exciting light with wavelength of 488 nm was utilized to verify the abnormal variation at lower temperature again.

**Figure 7:**
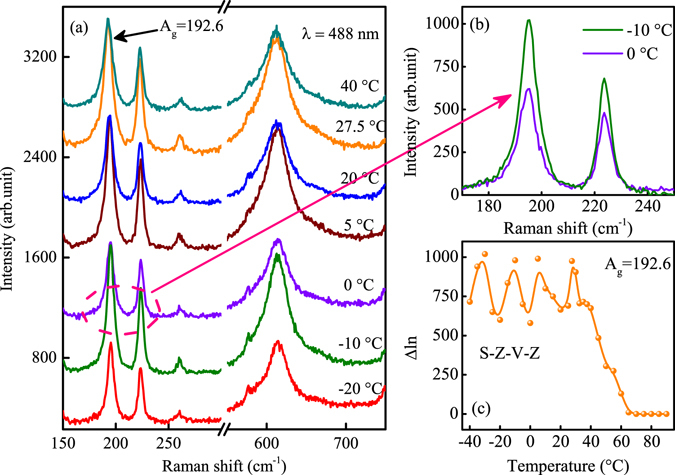
(**a**) The low temperature Raman spectra of the S-Z-V-Z structure, the wavelength of exciting light was 488 nm. (**b**) The enlarged picture of the Raman spectra with temperature of −10 °C and 0 °C, which was labeled by the pink dashed circle in (**a**). (**c**) The temperature dependent Raman intensity from low temperature to high temperature.

The low temperature Raman spectra of the S-Z-V-Z structure were shown in Fig. 7(a). It can be seen that the Raman intensity was reinforced greatly than that for the exciting light with wavelength of 633 nm. Obviously, the variation of the Raman spectra was similar to that in Fig. 5(d). It was found that a regularity of weaken-strengthen-weaken was followed by the Raman spectra. The comparison of the normalized Raman spectra with temperature of −10 °C and 0 °C was presented in Fig. 7(b). In order to show the variation of the Raman intensity clearly, the temperature dependent Raman intensity was extracted and shown in Fig. 7(c). From the picture, a periodical change can be seen in the temperature range from −40 °C to 40 °C. The variation period of the ΔIn is 20 °C. As we observed in Fig. 6(d) above, the ΔIn at 20 °C is corresponding to the valley of the period, which is in good agreement with that in Fig. 7(c). It seems that the double layer TCF plays a role of oscillator, the Raman vibrator of VO_2_ was limited in the oscillator and rocked periodicity at insulator state. The phenomenon is similar to the movement of the electron in the periodic potential field of lattice. Whether the periodic potential field can be formed between the two layer Al:ZnO, this will be further researched in our future work.

### Theoretical calculation

**Figure 8:**
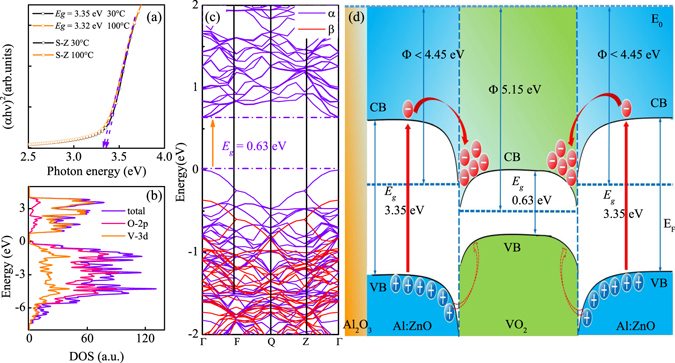
(**a**) The band gap of Al:ZnO obtained from the plot of (*αhν*)^1/2^ as a function of *hν*. (**b**,**c**) The total and partial electron density of states (DOS) of the VO_2_ film. (**d**) The energy band diagram of the *n-n* junction for the S-Z-V-Z.

Generally, the I type heterojunction will be formed in the interface when a material with broad band gap contacts with a narrow band gap material. Then, the narrow energy band will be embedded in the broad energy band and the electrons will be driven flow in the interface. Figure 8(a) shows the plot of (*αhν*)^1/2^ as a function of *hν*. The band gap of the Al:ZnO was extracted by intersect the *hν* axis through extended the vertical segment of the spectra. The *E*_*g*_ of the Al:ZnO is 3.35 and 3.32 eV for the 30 and 100 °C, respectively. The change of the *E*_*g*_ is only 0.03 eV. Therefore, the *E*_*g*_ of the Al:ZnO can be regarded as a constant in the range of 30–100 °C. The GGA + U (U = 5 eV) was adopted to calculate the electronic correlation and the band structure of VO_2_ with the Materials Studio 7.0 Package. (see detailed method in Supplementary Information S7 ^†^) Fig. 8(b) and (c) present the total and partial electron density of states (DOS) of the VO_2_ film. The band gap of VO_2_ is about 0.63 eV, consistent with other reports^43, 44^. In fact, the I type *n-n* junction can be formed once the VO_2_ and Al:ZnO contact.

### Electronic structures

According to the energy band theory of semiconductor, the electrons originating from the Al:ZnO will be injected to the conduction band of VO_2_ due to the potential energy difference. The extra electrons will add the threshold concentration of the phase transition of VO_2_. Therefore, the *T*_MIT_ can be reduced due to the electrons injection from the Al:ZnO layer. It is worthy noting that the ZnO layer acts as an electron transmission layer in solar cells, which also indicates the ZnO film enable provide electrons to VO_2_. The condition of the interface and the energy band diagrams of the *n-n* junction are shown in Fig. 8(d). The work function of VO_2_ and ZnO is 5.15 and 4.45 eV, respectively^45, 46^. Due to the heavily n-type doping of ZnO, the Fermi level of the Al:ZnO should be close to the bottom of conduction band. Therefore, the work function of Al:ZnO should be less than 4.45 eV due to the upper shift of the Fermi level. In order to avoid misunderstanding, the detailed value of the work function of Al:ZnO was not discussed here and presented by Φ < 4.45 eV in band alignment. The other energy band diagrams and the interface electrons transport images are shown in Supplementary Information S7. It should be emphasized that the effect of strain can be ignored, which was declared above. In the recent report, the effect of the Al doping was stated, from which the *T*_MIT_ can not be reduced to 68 °C^47^. Therefore, it can be believed that the decreased *T*_MIT_ of VO_2_ can be assigned to the electrons injection to the VO_2_ film.

### Hybrid VO_2_/Al:ZnO devices and electro-optic performance

**Figure 9:**
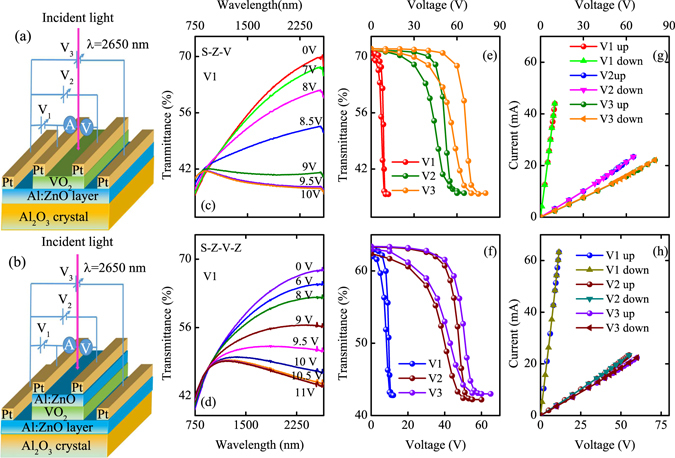
(**a**,**b**) The schematic shape of the S-Z-V and S-Z-V-Z heterojunctions devices, respectively. (**c**,**d**) The voltage dependent transmittance of the S-Z-V and S-Z-V-Z devices, respectively. (**e**,**f**) The hysteresis loop of the transmittance with different voltage at 2650 nm. (**g**,**h**) The I–V curves of the devices with different voltage.

The hybrid VO_2_/Al:ZnO heterojunctions devices were shown in Fig. 9(a) and (b). It can be seen that only single TCF layer was included in previous works, such as VO_2_/graphene, VO_2_/ITO systems. However, the configurations and effect of the double layer Al:ZnO are not reported previously. The voltage of V1, V2 and V3 was added in different electrodes, which were shown in the picture. Figure 9(c) and (d) present the optical transmittance of the two devices with different voltage of V1. One can see that the transmittance at infrared region decreases with the voltage. For the S-Z-V device, the voltage of transition (*V*_MIT_) is about 8.5 V and the saturation voltage is 9.5 V. However, it was found that the voltage, which leads to the Raman peaks disappear totally, is less than 9 V. This indicates that the Raman mode soften energy is lower than that for the overall transition energy. The voltage dependent Raman spectra for the S-Z-V was shown in Supplementary Information S8. The *V*_MIT_ and saturation voltage are 9 and 11 V for the S-Z-V-Z device, respectively. It can be seen that the *V*_MIT_ and saturation voltage of two layer Al:ZnO device are slightly higher than that for the one layer Al:ZnO device. For comparison, one can see the saturation voltages of V2 and V3 are much larger than that for V1. Noted that the electrode distance of V1 and V2 is about 300 *μ*m and 1 cm, respectively (Supplementary Information S9). The larger saturation voltages of V2 and V3 can be assigned to the larger area of the device (2 × 1 cm^2^) and electrode distance.

The hysteresis loop of voltage dependent transmittance at 2650 nm of the two devices are shown in Fig. 9(e) and (f). It can be seen that the saturation voltage is negatively correlated with the electrode distance. The current changes of the two devices are shown in Fig. 9(g) and (h). It should be noted that the current will change under the fluctuation of joule heating. However, the variation of the current is within 0.1 mA and has a subtle effect on the variation trend of I–V curves. Compared to the current of V1 for Z-V-Z-S device, the current is decreased by 20 mA for the S-Z-V while the current change of V2 and V3 for the two devices is nearly the same. The maximum current value of V2 and V3 for the two devices is about 23 mA. The phenomenon are specific and can not be ignored. Taken the joule heating into consideration, the joule heat was estimated and compared. According to the joule heat equation of Q = UIt, the joule heat is about 440 t, 1330 t and 1400 t for V1, V2 and V3 in the S-Z-V device, respectively. The joule heat for V1, V2 and V3 is 660 t, 1250 t and 1300 t in S-Z-V-Z device, respectively. Obviously, the joule heat for V1 is far less than that for V2 and V3 under the same time for the two devices. Considering the long horizontal distance of V2 and V3, the heat loss from the devices to the environment via thermal conduction should be considered. The great difference of the joule heating for the V2 and V3 from the V1 can be attributed to the larger surface area for the two devices. However, it can be seen that the joule heating for V1 in the S-Z-V device is less than that in the S-Z-V-Z device under the same electrode distance and the same device size. After careful analysis, we believed that the decrease of the joule heating may be assigned to the reduced heat loss or the effect of the electric field for V1 (not V2 and V3). Firstly, the heat loss in V1 may be more larger for the two layer Al:ZnO device than that for the one layer Al:ZnO device. Secondly, the horizontal distance of V1 is only 300 um for the two devices. The vertical distance of the electric field (50 nm) of V1 for the S-Z-V device is smaller than that (190 nm) for the S-Z-V-Z device. The electric field of V1 is more stronger for the S-Z-V device. Therefore, the lower voltage and joule heating was necessary for the MIT for the S-Z-V device. It is believed that the electric field could provide the help in regulating the MIT process from the response time of the switching behavior. This will be discussed in detail below.

**Figure 10:**
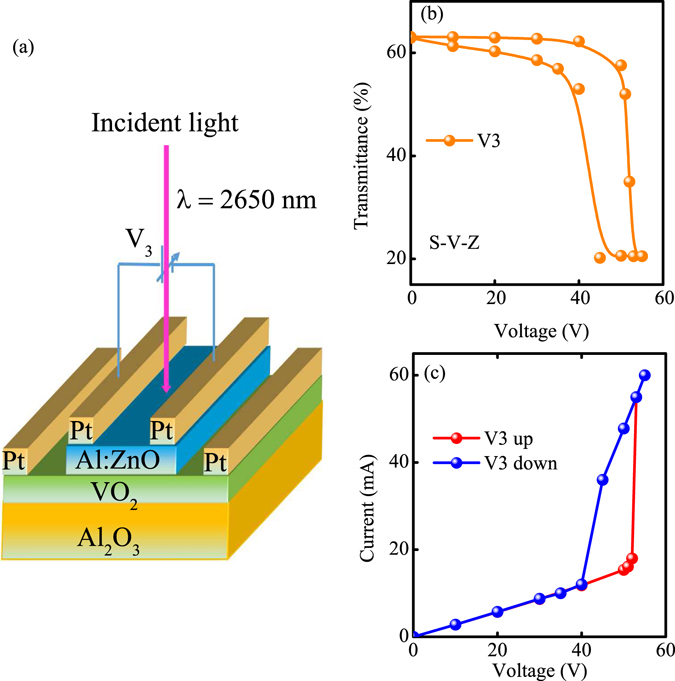
(**a**) The structure of the S-V-Z device. (**b**) The voltage dependent transmittance of the S-V-Z device. (**c**) The I–V curve of the device with V3.

The device of S-V-Z was shown in Fig. 10(a). The hysteresis loop of voltage (V3) dependent transmittance at 2650 nm was shown in Fig. 10(b) while the V1 and V2 dependent transmittance can be seen in supplementary information. It can be seen that the hysteresis loop of transmittance with V3 is similar to that for the devices of S-Z-V and S-Z-V-Z, while the V1 and V2 dependent transmittance is more sharper than that. Furthermore, the I–V curve is totally different from that for the S-Z-V and S-Z-V-Z devices, shown in Fig. 10(c). The I–V curve presented the hysteresis loop behavior rather than the simple direct proportion relationship. Similarly, the hysteresis behavior also can be found in the I–V curves for V1 and V2. We believed the sharp increase of the current is closely related to the hot carrier. In fact, the phenomenon is similar to the avalanche breakdown, which causes the current increasing abruptly. Comparing to the buffer layer of the Al:ZnO in the S-Z-V and S-Z-V-Z devices, the copping layer of the Al:ZnO in the S-V-Z making the I–V curve presents the hysteresis loop. The divergence result indicates that the influence of the Al:ZnO buffer and capping layer to the VO_2_ is different.

### The infrared response of the hybrid VO_2_/Al:ZnO heterojunctions devices

**Figure 11:**
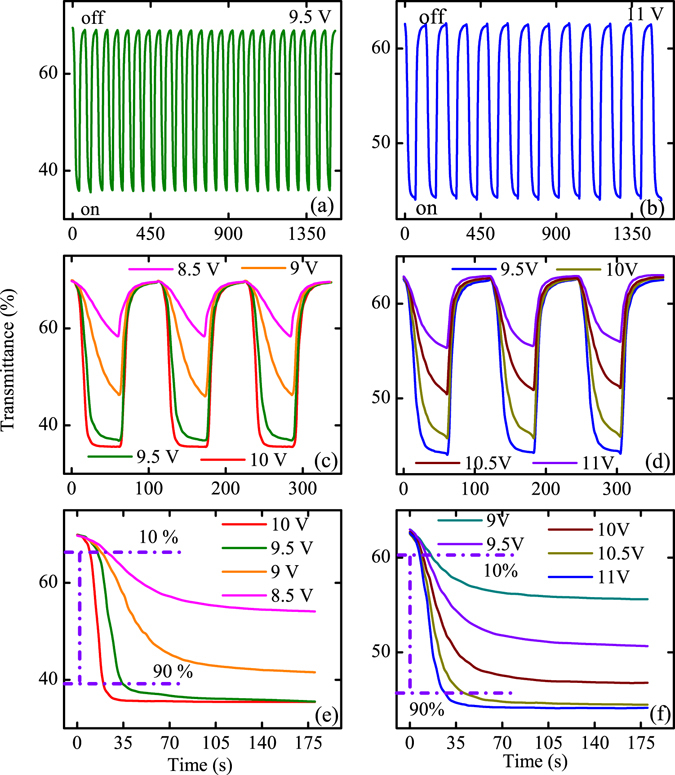
(**a**,**b**) The switching behavior from high transmittance to low transmittance when adding the saturation voltage for the two devices. (**c**,**d**) The switching performance of the devices for different voltage. (**e**,**f**) The transmittance response of enough voltage pulse duration for different voltage.

Figure 11(a) and (b) show the electro-optic switching behavior of the S-Z-V and S-Z-V-Z at 2650 nm. Though the switching results is similar to that reported by Li *et al*.^31, 48^ the key point is to compare the device performance in this study. Thanks to different VO_2_/ITO devices, the present study takes advantage of the vertical electrode to enhance the assistance effect of electric field. The pulse voltage between saturation voltage of V1 and 0 V was added. The pulse duration time is 35 s and 60 s for the S-Z-V and S-Z-V-Z device, respectively. For the S-Z-V device, the voltage pulse was switched between 0 V and 9.5 V while the switched voltage is between 0 V and 11 V for the S-Z-V-Z structure. It can be seen that the two devices show a continuous, pronounced and reproducible infrared response at 2650 nm. Note that there is nearly no transmittance loss in switch process, indicating the high quality and stability of the device. It is reported that the saturation of the two terminals devices based on the VO_2_/ITO is 15 V^31^. In this study, the saturation voltage of the V3 for the S-Z-V and S-Z-V-Z device is about 70 and 60 V (Fig. 9), which is larger than that for the VO_2_/ITO device. This can be attributed to the larger area of the devices (2 × 1 cm^2^) and long electrode distance. However, it can be seen that the saturation voltage of V1 for the S-Z-V and S-Z-V-Z is 9.5 and 11 V, which is smaller than that for the VO_2_/ITO device. The results indicate that the voltage can be reduced greatly through adopting the in-plant ladder-like electrode, which makes the heat loss greatly decreased.

Figure 11(c) and (d) present the switching behavior of the two devices with different voltage at infrared region (*λ* = 2650 nm). The voltage pulse duration is 60 s and the duty cycle is 0.5. One can see that the regulation rate is in direct proportion to the voltage and the recovery rate is unrelated to the input voltage. In order to compare the response time of the two devices, the enough voltage pulse duration and different voltage was added on the devices, which were shown in Fig. 11(e) and (f). In Fig. 11(e), the transmittance switching was totally completed at the input voltage of 10 V and 9.5 V and the response time from 10% to 90% switching is only 8 s and 18 s, respectively. However, it can be seen that the MIT cannot be completed by the input voltage of 9 and 8.5 V. Similarly, the 10% to 90% switching time is 19 s and 30 s under 11 V and 10.5 V voltage in Fig. 11(f), respectively. Due to the main effect of the joule heat, the response is slower than that for the micro devices. Li *et al*. takes advantage of the joule heating produced by Ag nanowires and ITO to induce the transition of the VO_2_ film^31, 48^. The infrared performance of the film can be dynamically regulated and controlled by an applied voltage successfully. However, it seems that the response time of the VO_2_/ITO device is slower than that in the present study for the V1. Therefore, it can be believed that adopting the in-plant ladder-like electrode may enhance the performance of VO_2_/Al:ZnO devices. Comparing the response time and transition voltage, it can be concluded that the overall performance of the S-Z-V device is the optimal among the S-V-Z, S-Z-V and S-Z-V-Z devices.

## Conclusion

In summary, the optical and electrical properties of the *n-n* type hybrid multilayer VO_2_/Al:ZnO heterojunctions have been investigated systematically. It was found that the TCF buffer and capping layer of Al:ZnO can provide electrons to VO_2_ film, through which the *T*_MIT_ of VO_2_ film can be reduced. From the temperature dependent reflectance, we found that the capping layer can be regarded as a highly absorptive dielectric film. The Fabry-Perot type cavities was formed by the VO_2_ film and Al:ZnO capping layer, which makes the valley of the reflectance red-shift with temperature in near infrared region. The red-shift of the valley can be ascribed to the refractive index change of the VO_2_ film. Unexpectedly, it was found that the Raman activity may be suppressed by the double layer Al:ZnO. By adding the external voltage on the ladder-like electrode devices, the switching behavior of the transmittance can be regulated and the infrared response is reproducible. Through comparing the response time and the regulated voltage, the electric field may provide the help in the manipulation of the switching behavior if the ladder-like electrode distance is small enough. It is believed that the results can be helpful in promoting the application of VO_2_/TCF devices.

## Methods

### Synthesis

The epitaxial Al:ZnO films were grown on non-annealed *c*-plane (0001) sapphire (Al_2_O_3_) substrate by PLD. The VO_2_ films were grown on the top or below of the Al:ZnO films. Further information about the experimental details are available in the Electronic Supplementary Information (ESI).

### Characterization

The crystal information on hybrid heterostructures was determined by X-ray diffraction with Cu *Kα* radiation (*λ* = 0.1542 nm). The component and valence state of the films were determinated by X-ray photoelectron spectroscopy (XPS, AXIS Ultra^DLD^, Japan) with Al *Kα* radiation (h*ν* = 1486.6 eV). The temperature dependent resistance was measured by THMSE 600 heating/cooling stage (Linkam Scientific Instruments). The surface morphology images and thickness of the films were acquired using atomic force microscopy (AFM: Digital Instruments Icon, Bruker) and scanning electron microscopy (SEM: Philips XL30FEG), respectively. The temperature-dependent transmittance spectra and near-normal incident (about 8°) reflectance spectra were measured by a double beam ultraviolet-infrared spectrophotometer (PerkinElmer Lambda 950) at the photon energy from 0.46 to 6.52 eV (190–2650 nm). The temperature-dependent Raman spectra were collected with a Jobin-Yvon LabRAM HR 800 micro-Raman spectrometer and a THMSE 600 heating/cooling stage (Linkam Scientific Instruments) in the temperature range from 20 °C to 90 °C. Laser with the wavelength of 633 nm and 488 nm was applied as the exciting light.

### Theoretical calculation

The theoretical calculation was based on the density function theory (DFT) within plane pseudopotentials and generalized gradient approximation (GGA) in the scheme of Perdew-Burke-Ernzerhof (PBE) exchange-correlation functional. An GGA + U (U is the Coulomb repulsion parameter) approach was utilized in the calculations. The on-site Coulomb correction applied to the 3d orbital electrons of V was taken as U = 5.0 eV and calculations were performed on a 2 × 2 × 2 VO_2_ supercell. The electron function was expanded in terms of plane-wave basis set with a cutoff energy of 400 eV and a Monkhorst-Pack k-point mesh of 2 × 2 × 2 VO_2_ was used for geometry optimization and property calculations. The convergence criterion of energy tolerance was 1.0 × 10^−6^ eV/atom. The maximal force, stress and displacement were 0.01 eV/Å, 0.02 GPa and 5.0 × 10^−4^ eV/Å.

## Electronic supplementary material

Supplementary Info File #1
