# Supplementary material for: The electro-optic mechanism and infrared switching dynamic of the hybrid multilayer VO2/Al:ZnO heterojunctions
Source: Sci Rep. 2017 Jun 30;7:4425. doi: 10.1038/s41598-017-04660-2 (PMC5493620; doi:10.1038/s41598-017-04660-2)
Supplement: Supplementary file 1 — Supplementary Info File #1 [file 41598_2017_4660_MOESM1_ESM.pdf]

Electronic Supplementary Information

The electro-optic mechanism and infrared switching dynamic of the hybrid multilayer VO_2/Al:ZnO heterojunctions

Peng Zhang[1], Wu Zhang[2], Junyong Wang[1], Kai Jiang[1], Jinzhong Zhang[1], Wenwu Li[1], Jiada Wu[2], Zhigao Hu[1,a)], and Junhao Chu[1]

[1]*Key Laboratory of Polar Materials and Devices (MOE) and Technical Center for Multifunctional Magneto-Optical Spectroscopy (Shanghai), Department of Electronic Engineering, East China Normal University, Shanghai 200241, China*

[2]*Department of Optical Science and Engineering, Fudan University, Shanghai 200433, China.*

[a)]Author to whom correspondence should be addressed.

Tel.: +86-21-54345150. Fax: +86-21-54345119.

Electronic mail: zghu@ee.ecnu.edu.cn

Film growth technique The VO_2 and Al:ZnO film was grown by pulsed laser deposition (PLD). In order to obtain better crystalline quality, the substrates were rigorously cleaned in pure ethanol with an ultrasonic bath and were rinsed several times by de-ionized water before the deposition. The VO_2 and Al:ZnO target were prepared from VO_2 powder (purity 99.95%) and Al:ZnO power. The VO_2 and Al:ZnO film were grown at room temperature and then annealed at 450° C in nitrogen ambience by a thermal process for 1 h. The detailed structure of the heterojunctions was shown in Figure S 1.

The cross section of the film Figure S 2 (a) and (b) show the high resolution scanning electron microscopy (SEM) pictures of the VO_2 and Al:ZnO film. It can be seen that the thickness of the film is about 50 and 140 nm for the VO_2 and Al:ZnO, respectively.

The XPS spectra of the VO_2 and Al:ZnO film The Figure S 3 (a) displays the overall core level XPS survey spectra of the VO_2 and Al:ZnO film. The intense peaks of the V 2p, Zn 2p and O 1s can be observed. Due to the doping of the Al is only 5%, the intensity of the Al peak is much weak, which can be seen in Figure S 3 (b). Figure S 3 (c) and (d) show the Lorentzian-Gaussian dividing peak analysis of V 2p and Zn 2p peaks for the VO_2 and Al:ZnO film, respectively. The 1/2 and 3/2 of the ZnO 2p peaks are found to be located at about 1021.7 eV and 1044.8 eV, respectively. From the V $2p_{2/3}$ peaks, it can be found that two peaks located at about 515.8 eV and 517.1 eV, which can be assigned to the oxidation state of V^{4+} and V^{5+}, respectively. The stoichiometry is about $VO_{1.95}$, which is consistent with our previous report. The O 1s peaks of the VO_2 and Al:ZnO film was shown in Figure S 2 (e) and (f). It was found that the main peak located at about 530.8 eV can be assigned to O 1s and no surface adsorption oxygen was detected in the Al:ZnO film. However, there is a distinct shoulder peak in the O 1s spectra for the VO_2 film, which means that the valence state of V^{4+} is tend to become V^{5+} in the air atmosphere.

Temperature dependent transmittance for the multilayer VO_2/Al:ZnO heterojunctions Figure S 4 (a)-(d) show the detailed temperature-dependent transmittance change of the four heterojunctions, from which the phase transition temperature can be obtained approximately. The saturation temperature of the transmittance is 90, 85, 85 and 80 ° C, which indicates the T_{MIT}

decreases with the number of Al:ZnO layer.

The hysteresis loops of temperature dependence transmittance and the differential curves The transmittance hysteresis loops at wavelength of 2650 nm were presented in Figure S 5 (a)-(d). It can be seen that the transition process just started for the S-V while the process for that is near the end for the S-Z-V-Z. This demonstrates that the Al:ZnO layer promoted the T_{MIT} decreasing. Figure S 5 (e)-(h) presented the related differential curves of the hysteresis loops. It is obvious that the T_{MIT} was reduced by 10 ° C for the S-Z-V-Z compared to that for the S-V.

Temperature dependent spectral of the Al:ZnO film The temperature dependent transmittance, reflectance and absorption spectra were shown in Figure S 6 (a)-(c). The variation of the spectra is subtle and can be ignored, which manifests the relevant physical phenomenon is caused by the change of VO_2 film.

The detailed theoretical calculation method, interface electrons transport pictures and energy level schematic The theoretical calculation was based on the density function theory (DFT) within plane pseudopotentials and generalized gradient approximation (GGA) in the scheme of Perdew-Burke-Ernzerhof (PBE) exchange-correlation functional. An GGA+U (U is the Coulomb repulsion parameter) approach was utilized in the calculations. The on-site Coulomb correction applied to the 3d orbital electrons of V was taken as U = 5.0 eV and calculations were performed on a $2 \times 2 \times 2$ VO_2 supercell. The electron function was expanded in terms of plane-wave basis set with a cutoff energy of 400 eV and a Monkhorst-Pack k-point mesh of $2 \times 2 \times 2$ VO_2 was used for geometry optimization and property calculations. The convergence criterion of energy tolerance was 1.0×10^{-6} eV/atom. The maximal force, stress and displacement were 0.01 eV/$\AA$, 0.02 GPa and 5.0×10^{-4} eV/$\AA$.

Figure S 7 (a) and (b) display the interface contact electrons transport condition of the S-V-Z and S-Z-V-Z. It can be seen that the electrons, which were from Al:ZnO film, were transported to the VO_2 film and the electron-hole pair was formed at the interface. The energy band diagram of the S-V-Z was shown in Figure S 7 (c).

Voltage dependent Raman spectra of the S-Z-V device Figure S 8 (a) and (b) display the voltage dependent Raman spectra of the S-Z-V device. It was shown that the Raman peaks is extremely weak at 8 V and totally disappeared at 9 V, which indicates that the saturation voltage is close to 8 V. From the voltage dependent transmittance, the saturation voltage is 9.5 V. It was found the saturation voltage for the Raman is lower by about 1 V than that for the transmittance. Note that the V_{MIT} of the transmittance is about 8.5 V. However, the Raman peaks nearly disappeared around the 8.5 V. The results demonstrate the transmittance transition is preceded by the lattice vibration soften.

The electro-optic performance of the S-V-Z device Figure S 9 (a) shows the S-V-Z device. It is worthy noting that the V3 is the voltage added on the Al:ZnO film rather than VO_2. The distance of the V1 electrode is about 300 um, which was shown in Figure S 9 (b). The voltage dependent transmittance was presented in Figure S 9 (c). It can be seen that the hysteresis loops of the transmittance is sharp than that for the other devices and the V_{MIT} is much large. We believed that the large voltage lead to the avalanche breakdown, which produced superfluous thermal and sharp phase transition. This can be reflected by the I-V curves in Figure S 9 (d). At a certain voltage, the current increased drastically, which means the occurrence of the avalanche breakdown. Therefore, the hysteresis loops of the transmittance and I-V curves is extremely sharp. However, for the voltage added on the Al:ZnO film (V3), the hysteresis loops of the transmittance and I-V curves is normal than that for V1 and V2. The picture can be seen in the article.

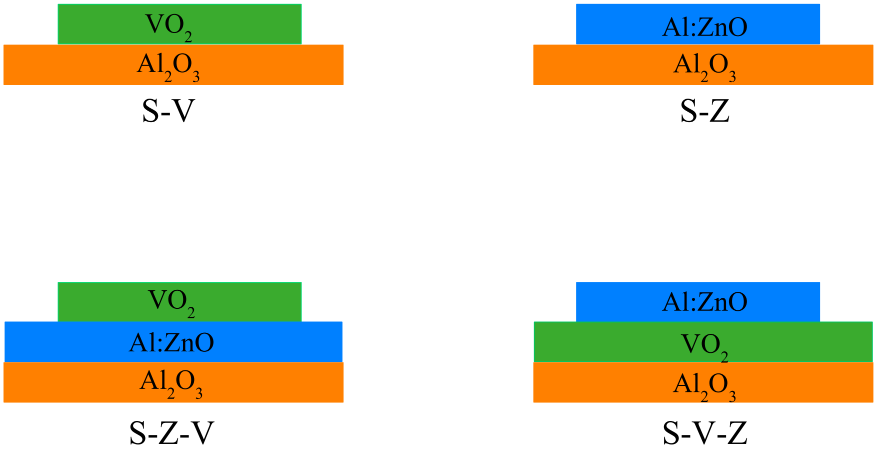

Fig. S 1: Schematic illustration of the hybrid multilayer VO_2/Al:ZnO heterojunctions.

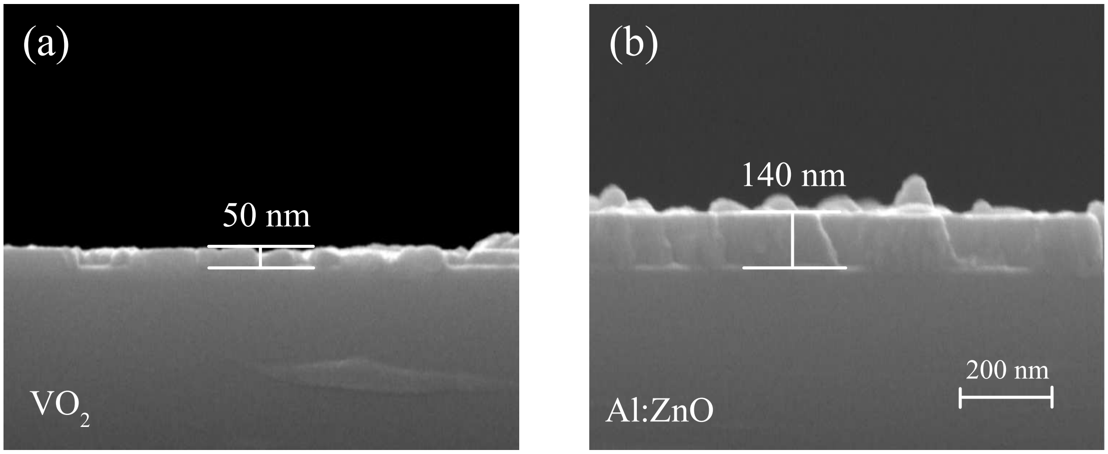

Fig. S 2: (a) and (b) The high resolution SEM pictures for the VO_2 and Al:ZnO film.

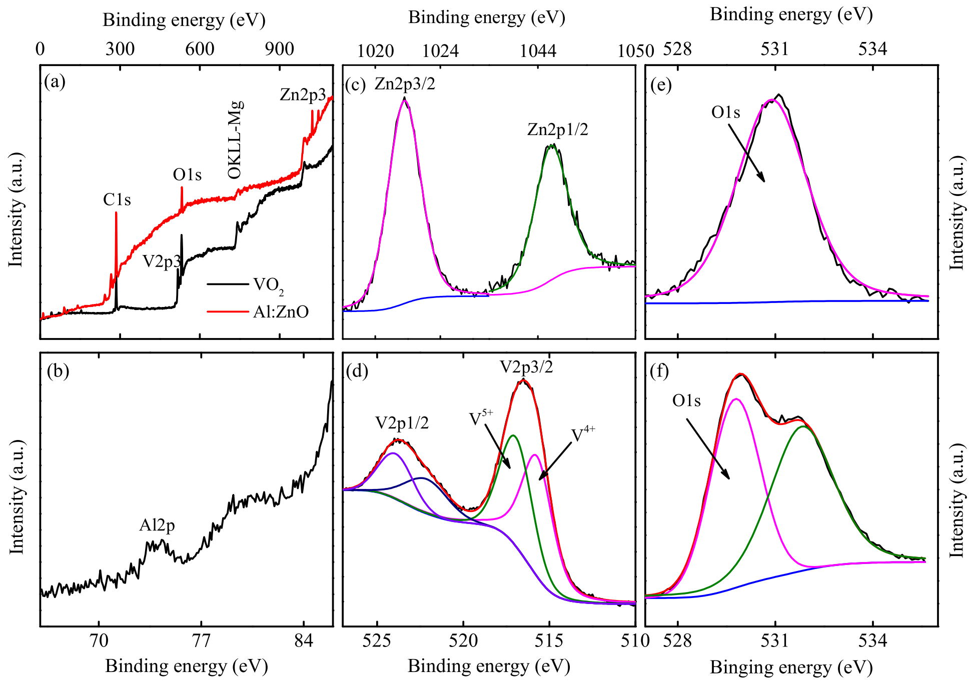

Fig. S 3: (a) Overall core level XPS spectra for the VO_2 and Al:ZnO film. (b) XPS spectra of the Al2p. (c)-(f) XPS spectra of V 2p lines and O 1s lines with the Lorentzian-Gaussian dividing peak analysis for the VO_2 and Al:ZnO film.

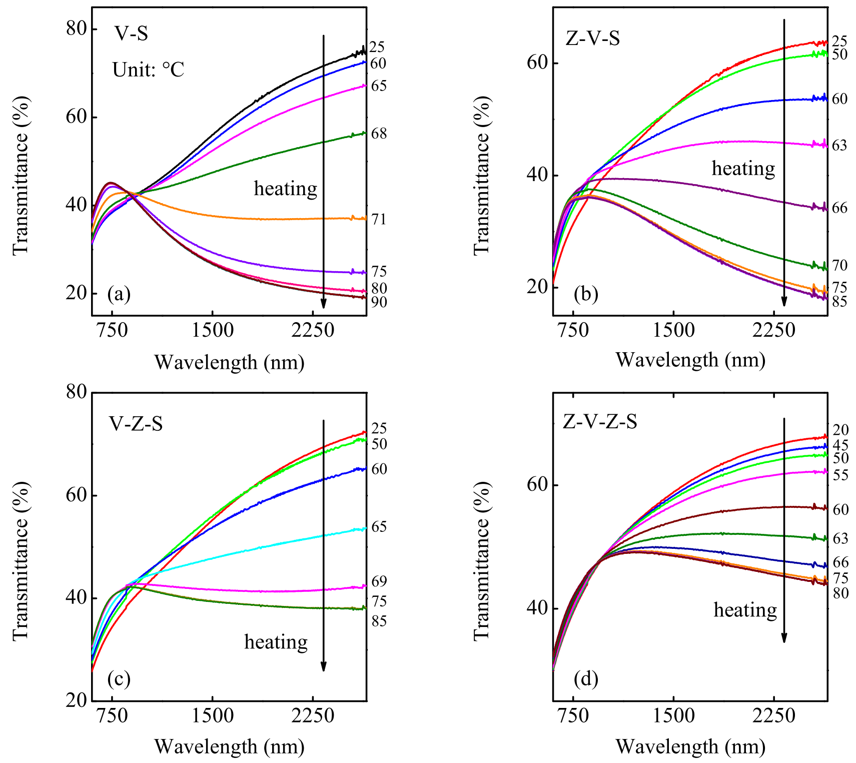

Fig. S 4: (a)-(d) Temperature dependent transmittance for the S-V, S-V-Z, S-Z-V and S-Z-V-Z.

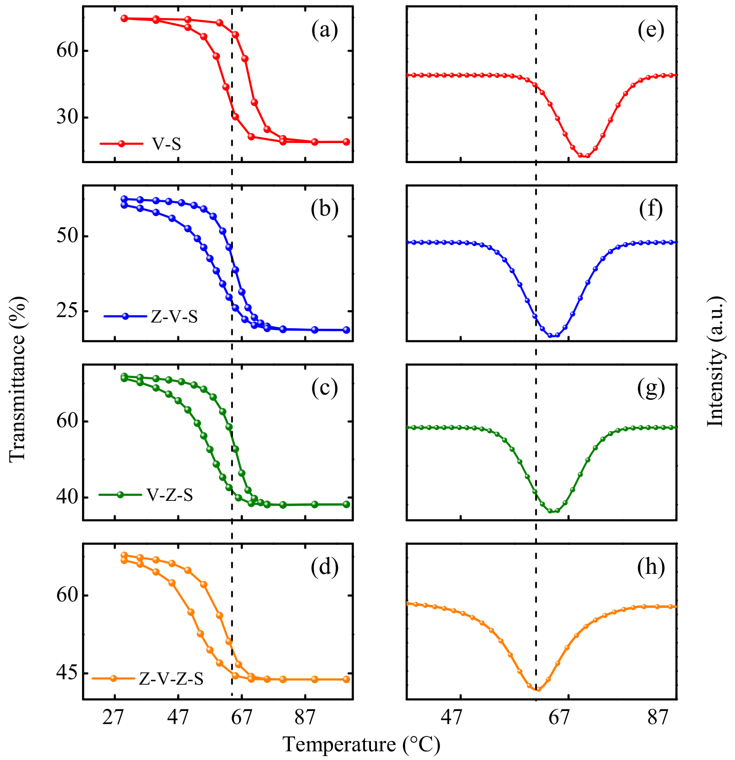

Fig. S 5: (a)-(d) The hysteresis loops for temperature dependence of transmittance at wavelength of 2650 nm. (e)-(h) The differential curves of the hysteresis loops.

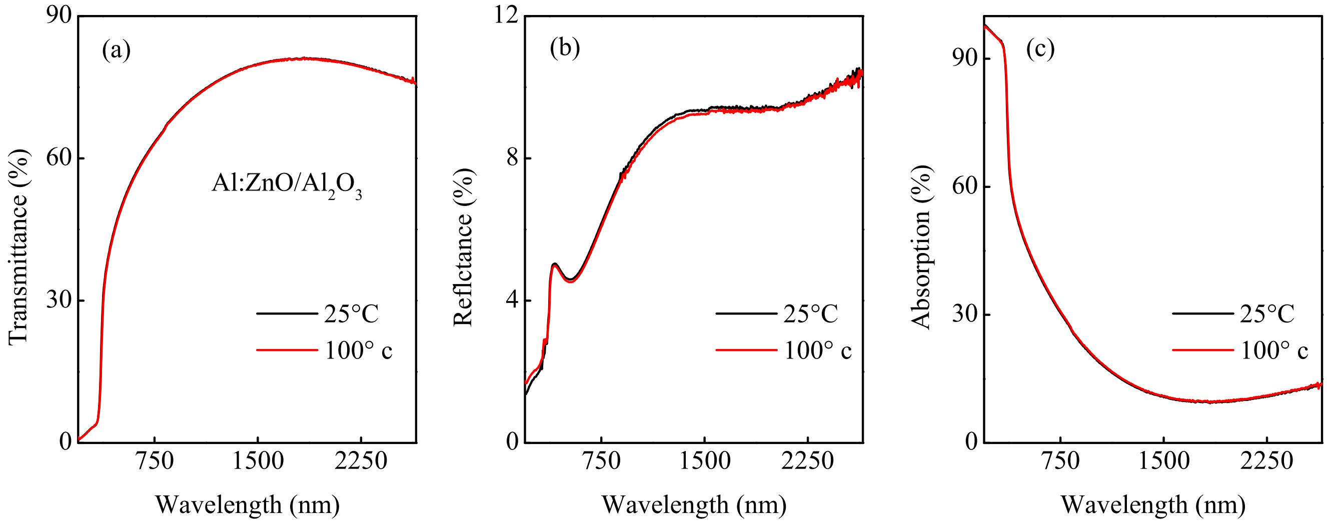

Fig. S 6: (a)-(c)Temperature dependent transmittance, reflectance and absorption spectra.

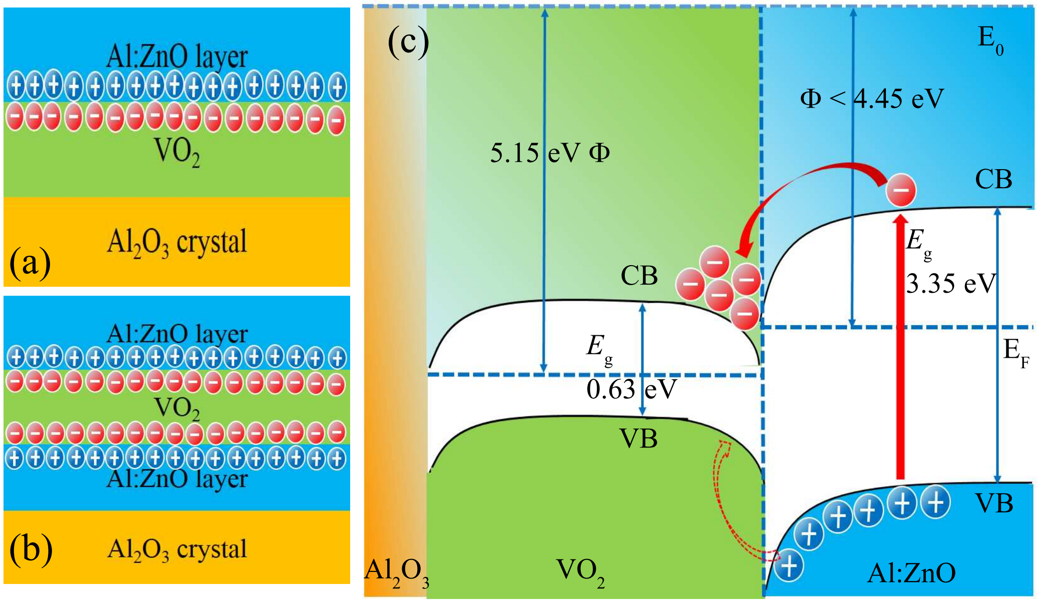

Fig. S 7: (a) and (d) The images of the interface electron transport. (c) The schematic energy band of the n-n S-V-Z heterojunction.

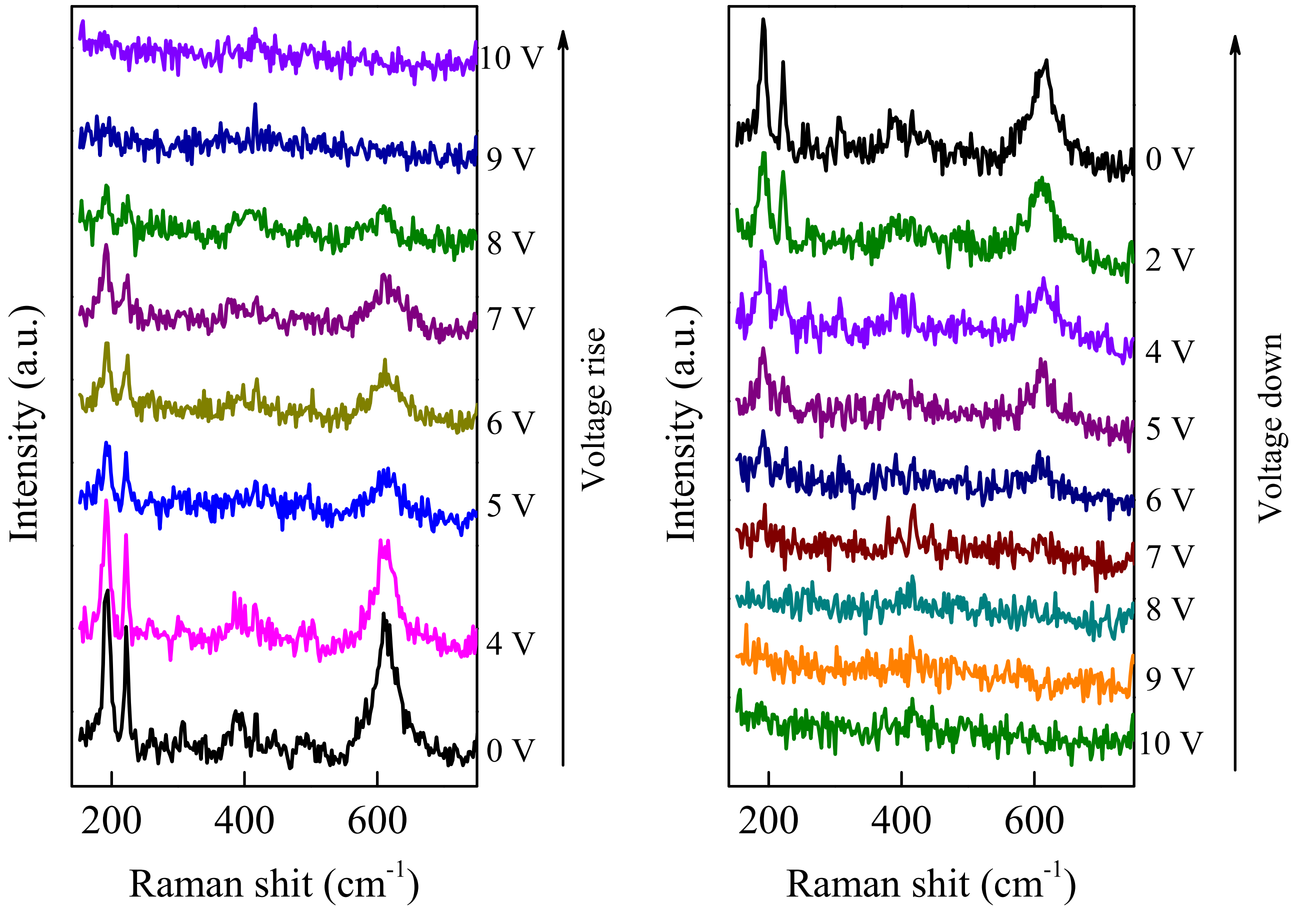

Fig. S 8: (a) and (b) The voltage dependent Raman spectra for the S-Z-V device.

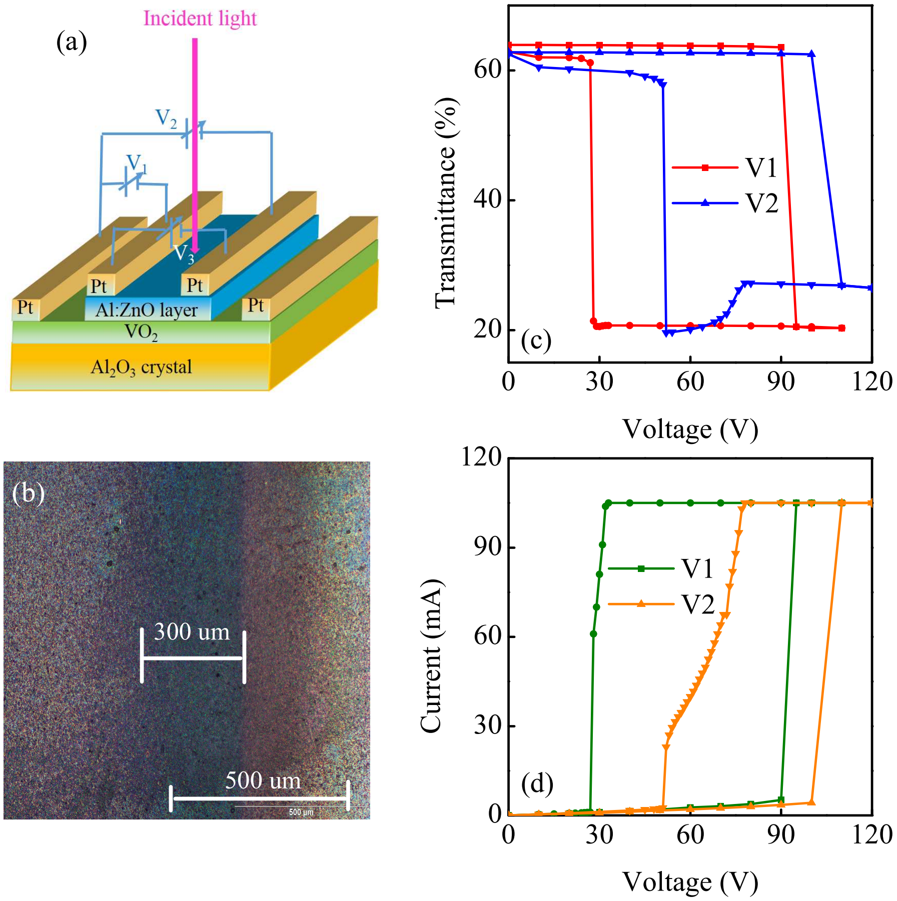

Fig. S 9: (a) The detailed structure of the S-V-Z device. (b) The distance of the electrode of V1. (c) and (d) The voltage dependent transmittance and the I-V curves for different voltage, respectively.
